# On the Difference Between Additive and Subtractive QM/MM Calculations

**DOI:** 10.3389/fchem.2018.00089

**Published:** 2018-04-03

**Authors:** Lili Cao, Ulf Ryde

**Affiliations:** Department of Theoretical Chemistry, Chemical Centre, Lund University, Lund, Sweden

**Keywords:** QM/MM, haem oxygenase, sulfite oxidase, mechanical embedding, electrostatic embedding, additive QM/MM, subtractive QM/MM

## Abstract

The combined quantum mechanical (QM) and molecular mechanical (MM) approach (QM/MM) is a popular method to study reactions in biochemical macromolecules. Even if the general procedure of using QM for a small, but interesting part of the system and MM for the rest is common to all approaches, the details of the implementations vary extensively, especially the treatment of the interface between the two systems. For example, QM/MM can use either additive or subtractive schemes, of which the former is often said to be preferable, although the two schemes are often mixed up with mechanical and electrostatic embedding. In this article, we clarify the similarities and differences of the two approaches. We show that inherently, the two approaches should be identical and in practice require the same sets of parameters. However, the subtractive scheme provides an opportunity to correct errors introduced by the truncation of the QM system, i.e., the link atoms, but such corrections require additional MM parameters for the QM system. We describe and test three types of link-atom correction, viz. for van der Waals, electrostatic, and bonded interactions. The calculations show that electrostatic and bonded link-atom corrections often give rise to problems in the geometries and energies. The van der Waals link-atom corrections are quite small and give results similar to a pure additive QM/MM scheme. Therefore, both approaches can be recommended.

## Introduction

Combined quantum mechanics and molecular mechanics (QM/MM) is a popular method to study biological macromolecules, as well as homogeneous catalysis and nanostructures, with computational methods (Balcells and Maseras, 2007; Lin and Truhlar, 2007; Ramos and Fernandes, 2008; Stoyanov et al., 2008; Senn and Thiel, 2009; Keal et al., 2011; Chung et al., 2015; Jover and Maseras, 2016; Ryde, 2016). In this approach, a small region of central interest (typically 20–300 atoms) is treated with quantum mechanical (QM) methods, whereas the remainder of the macromolecule, as well as a considerable amount of explicit solvent are treated by molecular mechanics (MM). This is supposed to combine the accuracy of QM methods with the speed of MM methods. Moreover, the entire macromolecule is included in the calculations [in contrast to the alternative QM-cluster approach (Blomberg et al., 2014), in which most parts of the macromolecule are omitted], reducing the risk of making a biased choice in the selection of the considered system and allowing for a detailed study of how the surroundings affect the properties of interest.

A problem with QM/MM approaches is that there exists so many variants and that the details of these are seldom discussed. For example, QM/MM approaches can use either subtractive or additive schemes (Senn and Thiel, 2009). In a subtractive scheme, three separate calculations are performed: One QM calculation with the QM region (system 1; $E_{1}^{\text{QM}}$) and two MM calculations, one for the entire system (systems 1 and 2; $E_{12}^{\text{MM}}$) and one for the QM region ($E_{1}^{\text{MM}}$) (Maseras and Morokuma, 1995; Ryde, 1996b; Svensson et al., 1996):

(1) $$\begin{array}{r} {\text{E}}_{\text{QM}/\text{MM}}^{\text{sub}}=\;{\text{E}}_{1}^{\text{QM}}+\;{\text{E}}_{12}^{\text{MM}}-\;{\text{E}}_{1}^{\text{MM}} \end{array}$$

The advantage with this approach is the simplicity: It automatically ensures that no interactions are double-counted and it can be set up for any QM and MM software (provided that they can write out energies and forces), without the need of any modification of the code. Thereby, the QM/MM software is updated every time the underlying QM or MM software is updated. Moreover, it can be easily extended to more than two computational methods and regions (Svensson et al., 1996). The typical example of a subtractive scheme is ONIOM (Svensson et al., 1996), but other software use similar methods, e.g., ComQum (Ryde, 1996b).

In the additive scheme, only two calculations are performed: the same QM calculation for the QM region, but only a single MM calculation ($E_{2-1}^{\text{MM}}$) (Sherwood et al., 2003; Senn and Thiel, 2009):

(2) $$\begin{array}{r} {\text{E}}_{\text{QM}/\text{MM}}^{\text{add}}=\;{\text{E}}_{1}^{\text{QM}}+\;{\text{E}}_{2-1}^{\text{MM}} \end{array}$$

although the latter is often formally divided into two terms, a MM energy of system 2 and a QM/MM interface energy ($E_{2-1}^{\text{MM}}=\;E_{2}^{\text{MM}}+\;E_{12}^{\text{QM/MM}}$). In this case, it is up to the developer to ensure that no interactions are omitted or double-counted. Therefore, an additive scheme requires a special MM software, in which the user or developer can select which MM terms to include. The advantage of the additive QM/MM scheme is that no MM parameters for the QM atoms are needed, because those energy terms are calculated by QM.

Further differences may arise if the QM region is covalently connected to the MM region. Then, the QM system needs to be properly truncated. This can be done by special localized orbitals (Levitt, 1976; Théry et al., 1994; Gao et al., 1998; Murphy et al., 2000; Senn and Thiel, 2009), but it is more common that the QM system is simply truncated by hydrogen atoms, the hydrogen link-atom approach (Singh and Kollman, 1986; Field et al., 1990; Reuter et al., 2000; Senn and Thiel, 2009; Ryde, 2016) In the subtractive scheme, MM parameters for the link atoms are needed (Senn and Thiel, 2009).

The interaction between the QM and MM regions is typically dominated by electrostatics. This interaction can also be treated at different levels of approximation (Senn and Thiel, 2009; Ryde, 2016). In mechanical embedding, it is calculated at the MM level (Maseras and Morokuma, 1995; Svensson et al., 1996). In electrostatic embedding, the electrostatic QM–MM interaction is instead treated at the QM level by including a point-charge model (i.e., atomic partial MM charges) of system 2 in the QM calculations (Singh and Kollman, 1986; Field et al., 1990; Ryde, 1996b; Dapprich et al., 1999). Thereby, system 1 is polarized by system 2, but not vice versa. In polarized embedding, both systems are mutually and self-consistently polarized in the QM calculations (Poulsen et al., 2001; Söderhjelm et al., 2009; Olsen et al., 2010). This requires a polarizable MM force field for system 2 (Lopes et al., 2009) and a QM software that can treat polarizabilities, which are still rather unusual. Therefore, such calculations are less common and typically restricted to single-point calculations of accurate properties. Mechanical embedding is normally considered to be less accurate than electrostatic embedding (Senn and Thiel, 2009), and the latter has therefore been the most widely used approximation, although it involves polarization of only parts of the system and is more sensitive to the treatment of the link atoms (Hu et al., 2011b). Strictly, Equations (1, 2) apply only to mechanical embedding, but they can easily be adapted to electrostatic embedding by including a point-charge model of system 2 in the QM term (*E*_QM1 + ptch2_) and setting the charges of system 1 to zero in the MM calculations (Ryde, 1996b).

Unfortunately, the distinction between the subtractive and additive schemes in literature is often unclear and confused. In many cases, the subtractive scheme is equated with mechanical embedding and the additive scheme with electrostatic embedding (Senn and Thiel, 2009; Götz et al., 2014). In other cases, the subtractive scheme is equated with the ONIOM method (Roßbach and Ochsenfeld, 2017). We prefer the definition in Equations (1, 2), emphasizing that the subtractive scheme employs two MM calculations with an external MM program, whereas the additive scheme employs a single MM calculation with an internal MM program, allowing the developer to cherry-pick the MM terms actually needed. In particular, both additive and subtractive schemes may use either mechanical or electrostatic (or even polarized) embedding.

It is normally assumed that the subtractive scheme is harder to set up and requires accurate MM parameters for the QM region and link atoms. For example, Roßbach and Ochsenfeld state in a recent article comparing subtractive and additive QM/MM (Roßbach and Ochsenfeld, 2017): “The (additive) QM/MM approach has the advantage that parameters for QM and link atoms, saturating covalent bonds between QM and MM, are unnecessary, as these are never described by the force field. The subtractive ONIOM approach requires accurate parameters for all atoms, including link atoms, because an MM calculation of the QM region is also necessary to avoid double counting.” On the other hand, Sousa et al. present the opposite view that the subtractive scheme is more advantageous, because of the “lack of a requirement for a parameterized expression describing the interaction of the various regions, and the fact that all systematic errors in the treatment of the inner regions by the lower levels of theory are canceled out” (Sousa et al., 2017).

In this article, we aim at clarifying the difference between the two schemes and compare their performance. We will show that with a proper setup, additive, and subtractive schemes should give identical results with a similar effort and that they require the same set of MM parameters. However, the subtractive scheme may be tuned to correct errors introduced by the link atoms and then additional parameters are needed.

## Methods

### The ComQum QM/MM software and its subtractive scheme

A problem when comparing QM/MM methods is that there exist so many variants and that the details of the calculations are seldom discussed (Ryde, 1996b; Senn and Thiel, 2009). Therefore, we here give a thorough discussion of our QM/MM software and details of all QM/MM variants implemented. All QM/MM calculations in this article were performed with the ComQum software (Ryde, 1996b; Ryde and Olsson, 2001). ComQum is a modular program, combining the QM software Turbomole (Ahlrichs et al., 1989; Furche et al., 2014; TURBOMOLE version 7.1, 2016) and the MM software AMBER (Case et al., 2014). It consists of five small Fortran programs that read, write, manipulate, and transfer coordinates, energies, forces, and charges between the MM and QM software. It employs a subtractive scheme and it was developed in 1992–1995, concurrently and independently from the ONIOM software (Ryde, 1995, 1996a,b). It has always used electrostatic embedding, in contrast to ONIOM. Junctions are treated by the hydrogen link-atom approach (Ryde, 1996b; Reuter et al., 2000; Senn and Thiel, 2009).

**Figure 1:**
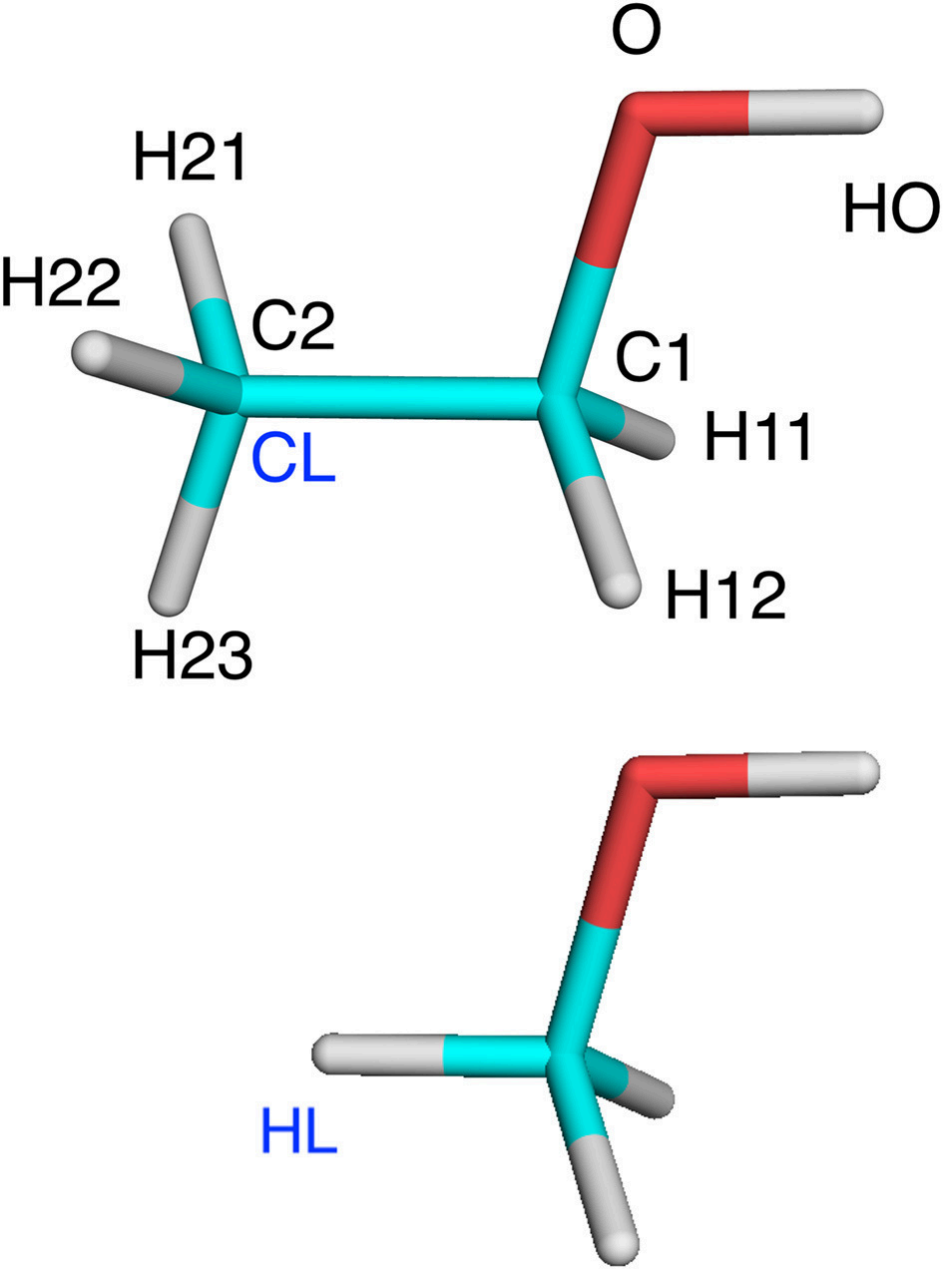
Ethanol and methanol with atom names indicated, as well as the CL and HL atoms.

To make the discussion clear, we use the following conventions (Senn and Thiel, 2009): The QM region is called system 1, whereas atoms in the MM region are called system 2. The QM region is terminated by hydrogen link atoms, called HL. They replace the corresponding carbon link (CL) atoms in the real system. HL and CL are different representations of the same atom and never appear in the same (MM or QM) calculation. This is illustrated in Figure 1. A superscript HL or CL show which representation is used. We will use XL to denote either HL or CL. The HL atom is covalently bound to a single Q_1_ atom the QM region, whereas the CL atom is connected to (typically several) M_2_ atoms in the MM region (we use this somewhat illogical notation, because several other QM/MM descriptions use M_1_ to denote CL; we prefer our notation because in ComQum, HL and CL are two different representations of the same atom, which belongs to the QM region). The Q_1_ atoms are covalently bound to Q_2_ atoms, which are covalently bound to Q_3_ atoms, and so on. Likewise, the M_2_ atoms are covalently bound to M_3_ atoms, and so on.

The HL atom is placed along the Q_1_−CL bond, with the Q_1_−HL bond length (*r*_*Q*_1_−*HL*_) calculated from (Ryde, 1996b):

(3) $$\begin{array}{r} r_{Q_{1}-\text{HL}}=r_{Q_{1}-\text{CL}}\frac{r_{Q_{1}-\text{HL}}^{0}}{r_{Q_{1}-\text{CL}}^{0}} \end{array}$$

where r_Q_1_−CL_ is the current Q_1_–CL bond length, ${\text{r}}_{{\text{Q}}_{1}-\text{CL}}^{0}$ is the optimum Q_1_–CL bond length in the MM force field and ${\text{r}}_{{\text{Q}}_{1}-\text{HL}}^{0}$ is the Q_1_–HL bond length in a model of the isolated truncated residue, optimized with the current QM method and basis set. ${\text{r}}_{{\text{Q}}_{1}-\text{CL}}^{0}$ can be found in the MM force field libraries and ${\text{r}}_{{\text{Q}}_{1}-\text{HL}}^{0}$is easily obtained by a simple QM geometry optimisation (which typically takes less than a minute). Equation (3) can also be used in reverse during the QM/MM geometry optimization to calculate the CL coordinates from the HL coordinates and it is also used together with the chain rule to obtain the MM forces on the HL atom (Ryde and Olsson, 2001). Thus, the HL atoms do not introduce any additional degrees of freedom.

The total QM/MM energy in standard ComQum (which uses a subtractive scheme with electrostatic embedding) is calculated from Equation (4) (Ryde, 1996b; Ryde and Olsson, 2001):

(4) $$\begin{array}{r} {\text{E}}_{\text{QM}/\text{MM}}^{\text{sub},\text{EE}}={\text{E}}_{\text{QM}1+\text{ptch}2}^{\text{HL}}+{\text{E}}_{\text{MM}12,{\text{q}}_{1}=0}^{\text{CL}}-E_{\text{MM}1,{\text{q}}_{1}=0}^{\text{HL}} \end{array}$$

where $E_{\text{QM1 + ptch2}}^{\text{HL}}$ is the QM energy of the QM system truncated by HL atoms and embedded in the set of point charges modeling system 2 (but excluding the self-energy of the point charges). All atoms in system 2 are included in the point-charge model (but not the CL atoms, which do not belong to system 2 in our view). In our original development, charges of some additional atoms were excluded (Ryde, 1996b), but a comparison of several different charge distribution schemes did not show any advantage of more complicated schemes (Hu et al., 2011b). $E_{\text{MM1},{\text{q}}_{\text{1}}=0}^{\text{HL}}$ is the MM energy of the QM system 1, still truncated by HL atoms, but without any electrostatic interactions. Finally, $E_{\text{MM12},{\text{q}}_{1}=0}^{\text{CL}}$ is the MM energy of all atoms in the system with CL atoms and with the charges of the QM system set to zero (to avoid double counting of the electrostatic interactions).

In the original implementation of ComQum (Ryde, 1996b), it was necessary to set up two AMBER parameter and topology (prmtop) files for the MM calculations, one for the full system ($E_{\text{MM}12,{\text{q}}_{1}=0}^{\text{CL}}$) and one for the truncated QM region ($E_{\text{MM}1,{\text{q}}_{1}=0}^{\text{HL}}$). This involved development of MM parameters for all junctions, which is tedious and error prone, even if the same truncated residues can be used for several different proteins. Initially, some attempts were made to allow the calculations on the small system compensate for the truncations and the introduction of link atoms (i.e., for the conversion of CL atoms to HL atoms). However, we did not see any consistent improvement in the results; instead such a treatment often introduced instability in the calculations.

In particular, we soon realized that the parameters of the HL–Q_1_ bond cannot be freely selected. The deterministic relation between HL and CL in ComQum (Equation 3) implies that we should use ${\text{r}}_{{\text{Q}}_{1}-\text{HL}}^{0}$ as the equilibrium bond length and the force constant must be

(5) $$\begin{array}{r} {\text{k}}_{{\text{Q}}_{1}-\text{HL}}={\text{k}}_{{\text{Q}}_{1}-\text{CL}}(\frac{{\text{r}}_{{\text{Q}}_{1}-\text{HL}}^{0}}{{\text{r}}_{{\text{Q}}_{1}-\text{CL}}^{0}}{)}^{2} \end{array}$$

where *k*_Q_1_−CL_ is the force constant of the Q_1_−CL bond in the MM force field. Otherwise, a spurious force will be introduced. Moreover, for the Q_2_−Q_1_−HL angle and the Q_3_−Q_2_−Q_1_−HL dihedral parameters we simply used the corresponding MM Q_2_−Q_1_−CL angle and the Q_3_−Q_2_−Q_1_−CL parameters, obtained by copying these entries from the MM force field.

Even if this was a completely mechanical procedure, it was still somewhat tedious and error prone, because it had to be redone every time the MM force field, QM method, or basis set was changed. Therefore, we implemented in 2006 a program (changeparm) that performed this task automatically: Reading the AMBER prmtop file of the full system and a file with the ideal QM bond length (${\text{r}}_{\text{XL}-\text{HL}}^{0}$), it automatically generates the prmtop and coordinate files for the MM calculation of the QM system ($E_{\text{MM}1,{\text{q}}_{1}=0}^{\text{HL}}$) according to these rules. Thereby, only a single MM calculation needs to be set up (that of the full system, which typically is already done, because QM/MM studies of macromolecules normally start with an equilibration of the structure with molecular dynamics) and no special parameters needs to be developed for the truncated system or the link atoms. This removes one of the disadvantages with the subtractive scheme (but it also discards a potential advantage with the method, as will be discussed below). However, the changeparm program required procedures to read and write the prmtop file, as well as a complete understanding of the meaning of all entries in it, a significant programming effort. Still, it has been so valuable that we recently have implemented the corresponding program (Cao et al., 2018) also for the crystallography and NMR system (CNS; Brunger et al., 1998; Brunger, 2007) software for quantum refinement (Ryde et al., 2002; Ryde and Nilsson, 2003).

Thus, with our implementation, also the subtractive QM/MM scheme requires only a single prmtop for the entire system. However, this file must contain parameters for all atoms, including those in the QM region. This is so because (the *leap* module in) AMBER refuses print the file if any parameter is missing. It may be that other MM software is less restrictive. However, this is a rather minor restriction, because the parameters do not need to be accurate. On the contrary, for all interactions involving only QM atoms (except van der Waals interactions involving the link atoms), the MM energies cancel exactly (owing to the $E_{\text{MM}12,{\text{q}}_{1}=0}^{\text{CL}}-E_{\text{MM}1,{\text{q}}_{1}=0}^{\text{HL}}$ terms in Equation 4). Therefore, dummy (e.g., zeroed) parameters may be used. Moreover, with the general MM force fields available in most MM software (Vanommeslaeghe et al., 2010), including AMBER (Wang et al., 2004), most parameters already exist. The only problem may be metal sites, but if no explicit bonds are defined between the metals and any ligand atoms, only van der Waals parameters for the metal are needed and these are necessary also in the additive scheme. Thus, the need of parameters for the QM region is a very minor restriction of the subtractive scheme and the setup of dummy parameters can easily be automatized (although we have never felt such a need).

### Additive comqum

Recently, we implemented a simple software (calcforce), which calculates MM energies and forces, based on AMBER coordinate and parameter–topology files. This was done to allow calculations without any cut-off for non-bonded interactions, even for very large systems (AMBER employs a non-bonded pair list that can become too large for the memory with more than ~10^5^ atoms) and to gain control over exactly what forces are written out by the program (for example, turning on the dumfrc option in the AMBER software changes the electrostatic energy). As a by-product, it also gives us full control over the energy function and allowed us to implement an additive QM/MM scheme.

In our additive approach, we use the energy function:

(6) $$\begin{array}{r} {\text{E}}_{\text{QM}/\text{MM}}^{\text{add},\text{EE}}={\text{E}}_{\text{QM}1+\text{ptch}2}^{\text{HL}}+{\text{E}}_{\text{MM }2-1}^{\text{CL}} \end{array}$$

where the $E_{\text{QM}1+\text{ptch}2}^{\text{HL}}$ QM term is identical to that used in the subtractive scheme. As shown by the superscript, all terms in $E_{\text{MM}\;2-1}^{\text{CL}}$ employ CL atoms, coordinates and parameters, never any HL atoms. We employed the following rules to determine what MM terms to include in $E_{\text{MM}\;2-1}^{\text{CL}}$ (note again that in our notation, the CL atoms belong to the QM region):

- All interactions involving only QM atoms are excluded (already included in the QM term).
- All interactions involving only MM atoms are included.
- Bonded interactions involving at least one MM atom are included.
- Electrostatic interactions involving one MM and one QM atom are excluded (already included in the QM calculation).
- Van der Waals interactions involving one MM and one QM atom are included.

These rules are based on the simple philosophy that we should calculate by MM all terms that are not already considered in the QM calculations. This seems very natural and should represent a typical implementation of additive QM/MM (Sherwood et al., 2003), although the rules are seldom discussed explicitly. This selection is illustrated in Table 1 for the simple ethanol model in Figure 1 (with CB as the link atom). Note that the first four rules are identical to the rules we use for setting up the truncated prmtop file in the subtractive scheme, so that the two schemes should give identical bonded and electrostatic energies. In particular, the two schemes give identical energies for the XL–Q_1_ bond term (assuming the harmonic term $E_{{\text{Q}}_{1}-\text{XL}}^{\text{bond}}={\text{k}}_{{\text{Q}}_{1}-\text{XL}}{\left({\text{r}}_{{\text{Q}}_{1}-\text{XL}}-\;{\text{r}}_{{\text{Q}}_{1}-\text{XL}}^{0}\right)}^{2}$ employed in AMBER and most other macromolecular force fields) even if the subtractive scheme uses HL coordinates and the additive scheme CL coordinates, because of the relations between the coordinates in Equation (3) and the force constants in Equation (5) (the force constants in Equation 5 were constructed with this aim).

**Table 1:** Illustration of which terms are included in the various energies for the ethanol molecules in Figure 1 (atom names are shown in that figure, except that H1 indicates either H11 or H12 and H2 indicates H21, H22, or H23).

	**$E_{\text{Q}\text{M}1+\text{ptch}2}^{\text{H}\text{L}}$**	**$E_{\text{M}\text{M}1,{\text{q}}_{1}=0}^{\text{H}\text{L}}$**	**$E_{\text{M}\text{M}1,\text{ptch}2}^{\text{H}\text{L}}$**	**$E_{\text{M}\text{M}12}^{\text{C}\text{L}}$**	**$E_{\text{M}\text{M }2-1}^{\text{C}\text{L}}$**
**BONDS**					
HO–O	QM	MM	MM	MM	
O–C1	QM	MM	MM	MM	
C1–H1	QM	MM	MM	MM	
C1–C2	QM,HL	MM,HL	MM,HL	MM,CL	
C2–H2				MM,CL	MM,CL
**ANGLES**					
HO–O–C1	QM	MM	MM	MM	
O–C1–H1	QM	MM	MM	MM	
O–C1–C2	QM,HL	MM,HL	MM,HL	MM,CL	
H1–C1–C2	QM,HL	MM,HL	MM,HL	MM,CL	
C1–C2–H2				MM,CL	MM,CL
**DIHEDRALS**					
HO–O–C1–H1	QM	MM	MM	MM	
HO–O–C1–C2	QM,HL	MM,HL	MM,HL	MM,CL	
O–C1–C2–H2				MM,CL	MM,CL
H1–C1–C2-H2				MM,CL	MM,CL
**NON-BONDED**					
HO–O	QM				
HO–C1	QM				
HO–H1	QM		Sc	Sc	Sc
HO–C2	QM,HL		Sc,HL	Sc,CL	Sc,CL
HO–H2	Ptch		Ptch	MM	MM
O–C1	QM				
O–H1	QM				
O–C2	QM,HL				
O–H2	Ptch		Ptch	Sc	Sc
C1–H1	QM				
C1–C2	QM,HL				
C1–H2	Ptch		Ptch		
H1–C2	QM,HL				
H1–H2	Ptch		Ptch	Sc	Sc
C2–H2	Ptch,HL		Ptch,HL		

*QM means that the term is included by the QM calculation, MM that it is included as a MM term, HL or CL that it is treated as a HL or CL atom, Sc that the non-bonded interaction is scaled down, and Ptch that it is treated by point charges*.

The only difference between the two schemes is the van der Waals interactions involving the link atoms and another atom in the QM system (possibly other link atoms): In the additive scheme, no such interactions are calculated by MM (because both atoms belong to the QM system). However, in the subtractive scheme, all van der Waals interactions involving link atoms are calculated twice: In $E_{\text{MM}12,{\text{q}}_{1}=0}^{\text{CL}}$, they are obtained with MM parameters and coordinates for CL atoms, whereas in $E_{\text{MM}1,{\text{q}}_{1}=0}^{\text{HL}}$, they are obtained with MM parameters and coordinates for HL atoms. In variance to all the other QM atoms, these two terms are not identical and therefore will not cancel. Instead, they provide a MM correction to the link atom, i.e., to the fact that the HL atoms in the QM region are H atoms and not the correct C atoms (i.e., they have smaller van der Waals radii) and that they are at incorrect positions. Thus, these van der Waals terms in $E_{\text{MM}1,{\text{q}}_{1}=0}^{\text{HL}}$ can be seen as a correction to the corresponding energy in the QM calculation ($E_{\text{QM}1+\text{ptch}2}^{\text{HL}}$), which also involves the incorrect HL coordinates and atoms. We will call it the *van der Waals link-atom correction* (VLAC). MM van der Waals parameters are normally quite accurate, so this approach is used in most subtractive schemes, but it cannot be included in a strict additive scheme, which may be a disadvantage. It should be noted that these interactions are only within the QM region, so with a small QM region, it involves only a few interactions and the correction is small.

In fact, we can exactly reproduce the additive QM/MM calculations within a subtractive scheme by replacing the $E_{\text{MM}1,{\text{q}}_{1}=0}^{\text{HL}}$ term in Equation (4) with a $E_{\text{MM}1,{\text{q}}_{1}=0}^{\text{CL}}$ term, in which parameters and coordinates corresponding to the CL atoms, rather than the HL atoms, are used. This was done manually to confirm that the implementations are correct, but it has never been implemented for production calculations (because the additive scheme gives the same results).

### Mechanical embedding

For comparison, we have also implemented mechanical embedding (ME) in ComQum [we have calculated single-point ME energies before (Hu et al., 2011a,b), but not done full geometry optimizations]. ME calculations can be run by simply deleting the point-charge model from the QM calculations (removing the $point_charges keyword from the Turbomole control file) and (re-)inserting charges of the QM system in the prmtop files for the two MM calculations in Equation (4). This gives the energy function:

(7) $$\begin{array}{r} {\text{E}}_{\text{QM}/\text{MM}}^{\text{sub},\text{ME}}={\text{E}}_{\text{QM}1}^{\text{HL}}+{\text{E}}_{\text{MM}12}^{\text{CL}}-{\text{E}}_{\text{MM}1}^{\text{HL}} \end{array}$$

Two issues need to be settled in this implementation. The first is how to treat the link atoms. If the charge of the HL atom is identical to that of the CL atom, the electrostatics within the QM system cancel exactly in the $E_{\text{MM}12}^{\text{CL}}-E_{\text{MM}1}^{\text{HL}}$ terms in Equation (7). However, then the total energy reflects electrostatics involving HL atoms, from the $E_{\text{QM1}}^{\text{HL}}$ term. In analogy with the van der Waals energy correction described in the previous section, we prefer to have different charges for the HL and CL atoms, with those of the HL atoms being representative for a H atom and those of the CL atoms being representative for the true (typically C) atoms.

This leads us to the second issue, viz. how the charges are calculated for the QM system, including the HL and CL atoms. Since QM calculations are done for the QM system, it is natural to use some sort of QM-derived charges. Originally, ComQum employed Mulliken charges (these charges are used also with electrostatic embedding when parts of system 2 is optimized by MM; Ryde, 1996b). However, it is well-known that charges fitted to the electrostatic potential (ESP) give (by construction) more accurate electrostatic interaction energies (Sigfridsson and Ryde, 1998). Therefore, we have used such ESP charges [obtained with the Merz–Kollman scheme (Besler et al., 1990)] ever since ESP charges were implemented in Turbomole (note that in Turbomole, the point-charge model needs to be removed before the charges are calculated, without reoptimizing the wavefunction). These charges can be directly used in the $E_{\text{MM}1}^{\text{HL}}$ term and therefore also for the HL atoms, since they were obtained for a QM system with HL atoms.

However, for the $E_{\text{MM}12}^{\text{CL}}$ term, these charges need to be adapted so that the charge of the total system remains integer, meaning that the HL charges need to be adapted to apply for CL atoms instead. It is not evident how this should be done and it is seldom discussed, although this needs to be done for essentially all QM/MM and MD simulations employing QM charges calculated for QM systems with link atoms.

We have selected to follow this procedure:

1. Start from the MM charges of the entire system with formal (integer) charges on metals and other inorganic groups.
2. Use the QM charges for all QM atoms, except the HL atoms.
3. Use the original MM charges for all MM atoms.
4. For each amino acid (or other biochemical building unit) with link atoms: Add a constant offset to the original MM charges of each CL atom in the unit so that the total charge of this unit is the same as the sum of the QM charge of these atoms, possibly with the addition of an integer charge from atoms outside the QM system.

The procedure is illustrated in Table 2. It is fully automatic, except that a possible integer charge outside the QM system needs to be specified. This way, charge transfer within the QM system is allowed, meaning that none of the QM residues has an integer charge. The modification of the charges on the CL atoms is also kept to a minimum. However, alternative approaches are conceivable, e.g., dividing the remaining charge equally over all link atom, after the charges of the non-HL QM atoms have been set. Thus, the CL charges are somewhat ambiguous. Importantly, the procedure keeps all charges of the QM system equal between $E_{\text{MM}12}^{\text{CL}}$ and $E_{\text{MM}1}^{\text{HL}}$, except for the HL/CL atoms, allowing for a proper cancelation of those electrostatic terms in the ME approach, in analogy with the VLAC correction for the subtractive scheme.

**Table 2:** Illustration of the method to determine charges for methanol and ethanol (shown in Figure 1 with atom names; H1 is H11 and H12; H2 is H21, H22, and H23).

	**Methanol**	**Ethanol**	
**Atom**		**Set1**	**Set2**
C2	−0.0108	−0.2324	−0.1935
H2		***0.0609***	***0.0609***
C1	**0.2120**	0.3841	**0.2120**
H1	−**0.0043**	−0.0534	−**0.0043**
O	−**0.5903**	−0.5847	−**0.5903**
HO	**0.3977**	0.3570	**0.3977**

*The methanol charges were obtained from a QM RESP calculation. The ethanol Set1 charges were obtained in the same way. For Set2, charges of the C1, H1, O, and H atoms were taken from methanol (marked in bold face) and the charges of H2 were taken from Set1 (marked in bold face and italics). Finally, the charge on C2 was determined to give a vanishing net charge*.

### Electrostatic link-atom corrections

In the electrostatic-embedding variant of ONIOM (Vreven et al., 2006), as well as in our QTCP approach (QM/MM thermodynamic cycle perturbation; Rod and Ryde, 2005), further attempts are made to correct errors introduced by the link atoms. In the QM calculations, the HL atoms are of the wrong element, located at the incorrect position (compared to the CL atom) and they may make Coulombic interactions with point charges of nearby atoms that are not included or are scaled down in normal MM calculations (viz. interactions with the M_2_, M_3_, and M_4_ atoms). These errors can be compensated by calculating exactly the same interactions in the $E_{\text{MM}1}^{\text{HL}}$ term and replace them with the corresponding interactions in the $E_{\text{MM}12}^{\text{CL}}$ term.

In practice, this is accomplished by using QM charges for the QM system in both MM calculations and including the same point-charge model of the surroundings in the $E_{\text{MM}1}^{\text{HL}}$ term. We call this approach electrostatic link-atom correction (ELAC) and it gives the following energy function:

(8) $$\begin{array}{r} {\text{E}}_{\text{QM}/\text{MM}}^{\text{sub},\text{ELAC}}={\text{E}}_{\text{QM}1+\text{ptch}2}^{\text{HL}}+{\text{E}}_{\text{MM}12}^{\text{CL}}-{\text{E}}_{\text{MM}1+\text{ptch}2}^{\text{HL}} \end{array}$$

For these calculations, we used the same QM charges for the QM system and the same MM charges for MM system as described for the mechanical-embedding calculations.

### Bonded link-atom corrections

Finally, the subtractive scheme allows for a third type of link-atom corrections, viz. for the bonded terms. It is likely that a HL atom will give rise to slightly different bonded terms than the corresponding CL atom, e.g., smaller XL–Q_1_−Q_2_ ideal angles. Again, we may try to use the $E_{\text{MM}1}^{\text{HL}}$ calculation to correct for these errors (i.e., so that the HL bonded terms would cancel between the $E_{\text{QM}1+\text{ptch}2}^{\text{HL}}$ and $E_{\text{MM}1}^{\text{HL}}$ terms and the corresponding CL result in $E_{\text{MM}12}^{\text{CL}}$ would remain, instead of the exact cancelation of these terms between $E_{\text{MM}1}^{\text{HL}}$ and $E_{\text{MM}12}^{\text{CL}}$ as in both the standard additive and subtractive approaches). This is done by using different parameters for the Q_2_−Q_1_–HL and Q_2_−Q_1_–CL angles and the Q_3_−Q_2_–Q_1_−HL and Q_3_–Q_2_−Q_1_−CL dihedral parameters (and possibly also for the Q_1_–HL and Q_1_–CL bond parameters). We will call this bonded link-atom corrections (BLAC).

Of course, the parameters need to be accurate for there to be a hope of any improved results. In this paper, we tried two approaches. In the first (BLAC1), we used standard AMBER parameters for both the CL and HL terms, the latter typically coming from the GAFF (Wang et al., 2004) force field. These parameters involved also the bonded Q_1_-XL terms.

In the second approach (BLAC2), we instead performed a parametrisation of both the full and truncated systems, based on a QM frequency calculation on each system. The bonded parameters were then extracted with the Seminario approach (Seminario, 1996), using the Hess2FF program (Nilsson et al., 2003; Hu and Ryde, 2011). We used the same atom types as in the AMBER files in BLAC1. This means that in principle all bonded parameters will differ in the two MM calculations, not only those involving the XL atom. Finally, BLAC1J and BLAC2J was obtained from BLAC1 and BLAC2 by changing the Q_1_–HL force constants according to Equation (5), keeping everything else the same.

### Test systems

All QM calculations were carried out using the Turbomole software (versions 7.1 and 7.2; Ahlrichs et al., 1989; Furche et al., 2014). They were performed using the TPSS (Tao et al., 2003) functional in combination with def2-SV(P) (Schäfer et al., 1992) basis set, including empirical dispersion corrections with the DFT-D3 approach (Grimme et al., 2010) with Becke–Johnson damping (Grimme et al., 2011), as implemented in Turbomole. The MM calculations were performed with the AMBER ff14SB (Maier et al., 2015) force field for protein residues, GAFF (Wang et al., 2004) for non-protein molecules and TIP3P (Jorgensen et al., 1983) for water. In all QM/MM calculations, the MM system was kept fixed to simplify the interpretation of the results.

The various approaches were tested on three systems. The first was an isolated ethanol molecule. The reference system was ethanol, optimized with QM. In the QM/MM calculations, the QM system was methanol with C2 converted to a HL atom (Figure 1). The terminal methyl group was in the MM system.

**Figure 2:**
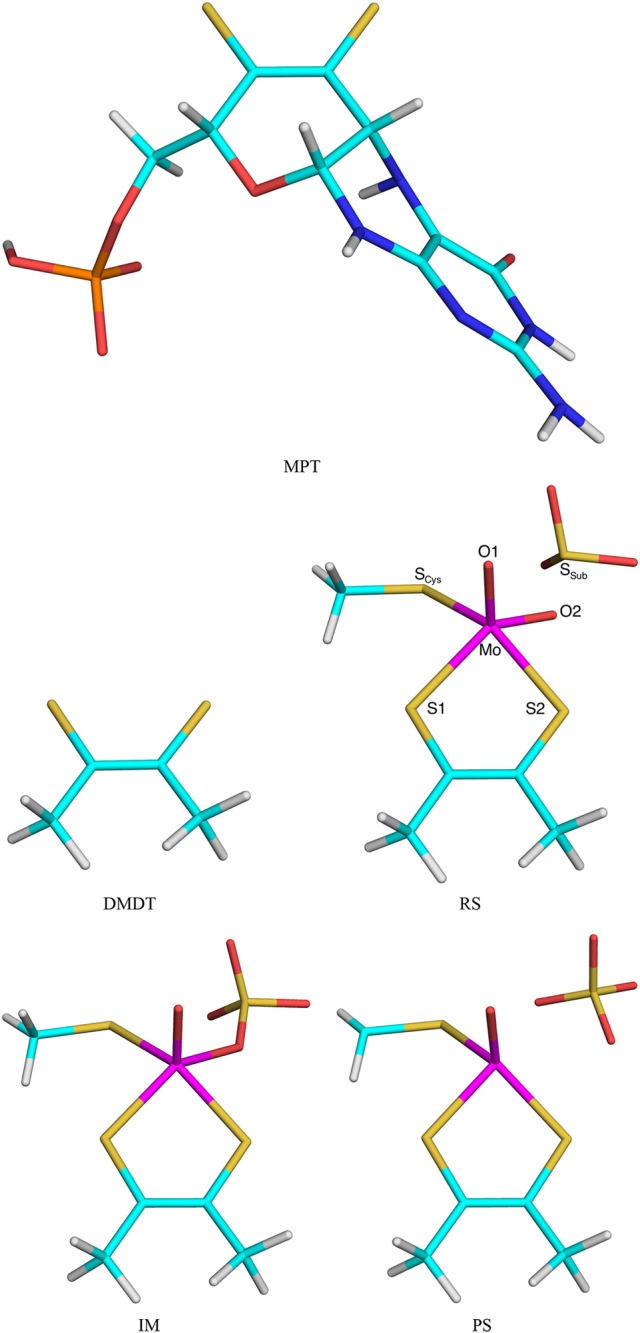
The MPT and DMDT ligand, as well as the RS, Im, and PS states in the reaction mechanism of sulfite oxidase. Atom names are indicated for the RS.

The second test system was sulfite oxidase. The calculations were taken from our recent study of this enzyme (Caldararu et al., 2018). The active site contains a Mo ion coordinated to a molybdopterin (MPT) molecule, as well as a Cys residue and two oxo groups. All these groups were included in the QM system (Cys modeled as CH_3_S^−^), as well as the sulfite substrate [note that this QM system is smaller than in our previous study (Caldararu et al., 2018), in which nine additional residues and five water molecules were also included]. In this paper we compared the effects of either including the full MPT residue (in the reduced and protonated form, called MPH in our previous paper) or truncating it to a dimethyldithiolene molecule [DMDT, (CH_3_CS${(}_{2}^{2-}$; both shown in Figure 2]. Thus, the QM/MM calculations with the full MPT molecule were the reference structures and QM/MM calculations with DMDT in the QM system were run to compare the performance of the various QM/MM variants.

In DMDT two of the CL atoms are covalently bonded. This gives a complication when automatically setting up the prmtop file for the truncated system: This bond needs to be explicitly removed from the file (together with the corresponding angles and dihedrals); otherwise, spurious MM forces will cause the calculations to crash with distorted structures. Normally, we discourage from having covalently connected CL atoms, but in this study, it provides a hard test for the various methods to provide junction corrections.

Five states were studied in the reaction: The Mo^VI^ = O+${\text{SO}}_{3}^{2-}$ reactant state (RS) with sulfite in the second coordination sphere of Mo, the Mo^IV^-${\text{SO}}_{4}^{2-}$ intermediate (Im) with sulfate coordinated to Mo, the Mo^IV^+${\text{SO}}_{4}^{2-}$ product state (PS) with sulfate in the second sphere of Mo (shown in Figure 2), as well as the two transition states (TS1 and TS2) connecting these three states. The transition states were obtained from potential-energy scans along the S–O1 (TS1, 2.0 Å) and Mo–O1 (TS2, 3.7 Å) reaction coordinates. PS was also obtained with a restraint in the Mo–O2 distance of 3.85 Å, taken from calculations with an appreciably larger QM system (Caldararu et al., 2018). Besides the QM system, the setup was identical to that in our previous study (Caldararu et al., 2018).

**Figure 3:**
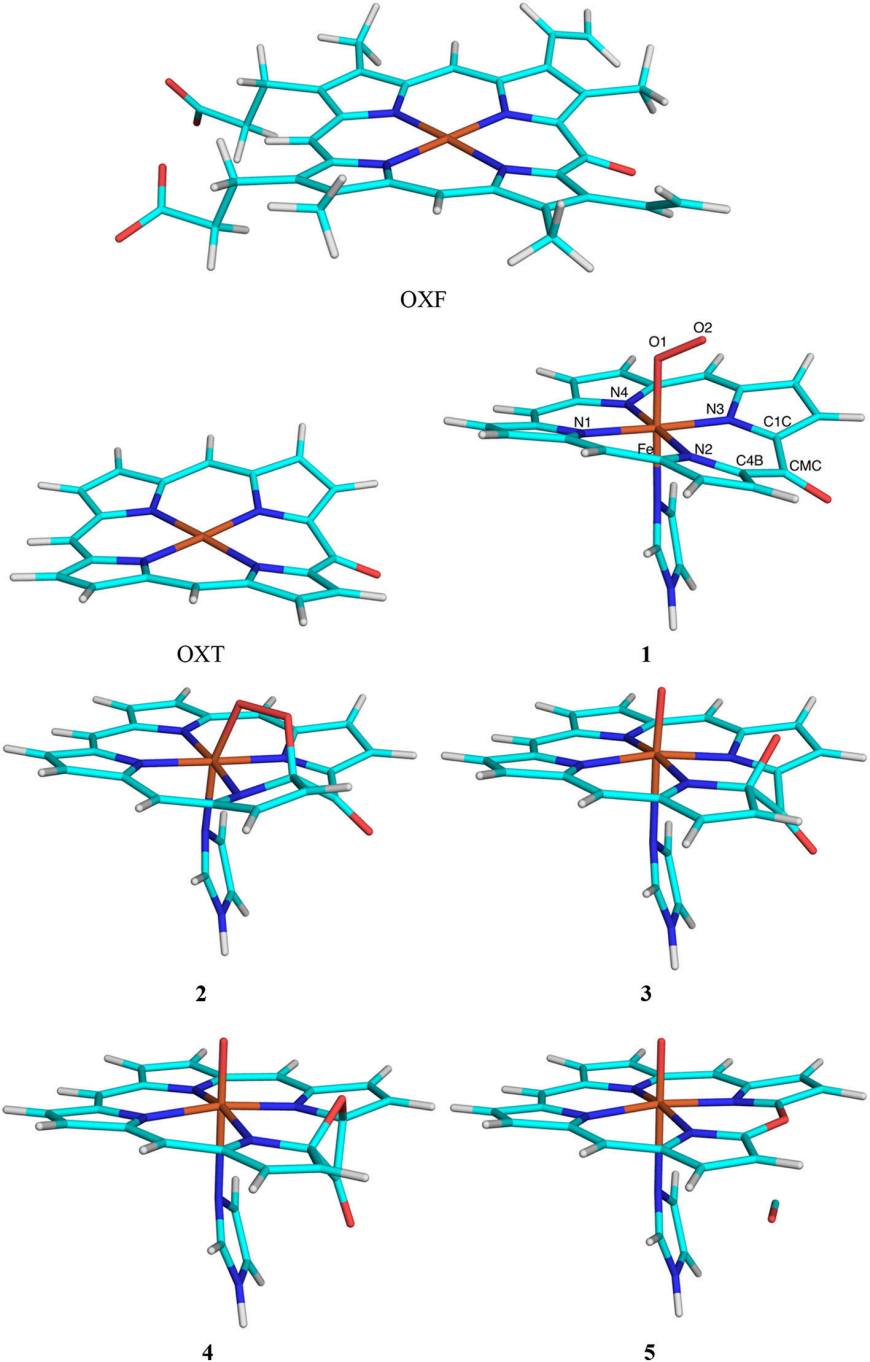
The OXF and OXT ligands, as well as the five states in the reaction mechanism of haem oxygenase. Atom names are indicated for the state **1**.

The third test system was the conversion of oxophlorin to verdohaem by haem oxygenase. Again, the calculations were taken from a recent study of this enzyme (Alavi et al., 2017). The QM system consisted of the oxophlorin group (an oxidized haem molecule), as well as O_2_ and His (truncated to imidazole) as axial ligands of the Fe ion [again, this QM system is smaller than in our previous study (Alavi et al., 2017), in which two additional residues and six water molecules were also included]. We compared the effects of including either the full oxophlorin ring (OXF) with its eight peripheral propionate, vinyl and methyl substituents or truncating all substituents to HL atoms (OXT; both shown in Figure 3). Thus, the calculations with the full OXF were the reference structures and QM/MM calculations with OXT were run to compare the performance of the various QM/MM variants.

Nine states were studied in the reaction as is shown in Figure 3: The Fe–O_2_ reactant state (**1**), the first intermediate, in which O_2_ is bridging between Fe and one of the OXF carbon atoms (C4B; **2**), the second intermediate (**3**) in which the O1–O2 bond is cleaved and the C4B–O2 bond is formed, the third intermediate (**4**) in which the O2 atom has formed a bond with the C1C OXF atom, giving a four-membered C4B–O2–C1B–CMC ring, the verdohaem product (**5**), in which CO has dissociated, but the C4B–O2–C1B bonds are kept, as well as the four connecting transition states (**T1**–**T4**). The transition states were obtained from potential-energy scans along the O2–C4B (**T1**, 1.8 Å), O1–O2 (**T2**, 1.6 Å), O2–C1B (**T3**, 1.9 Å), and the C4B–C (**T4** 1.8 Å). Besides the QM system, the setup was identical to that in our previous study (Alavi et al., 2017).

## Result and discussion

In this paper, we clarify the difference between additive and subtractive variants of the QM/MM approach. In principle, the two approaches can be tuned to give exactly the same results, as the additive approach can freely pick almost any energy in the $E_{2-1}^{\text{MM}}$ term in Equation (2). However, in a typical implementation of the two approaches, the primary difference between the two approaches is that the additive scheme employs only a single MM term for each interaction, whereas in the subtractive scheme, there are two MM terms for the QM system, one in $E_{12}^{\text{MM}}$ and one in $E_{1}^{\text{MM}}$. Depending on the implementation, these duplicate terms can either be selected to be identical (and therefore canceling, which would give exactly the same results as in the additive scheme) or they can be different, in particular with the aim of correcting the errors introduced by the HL link atoms in the QM system. We have investigated three different levels of link-atom corrections, involving van der Waals terms (VLAC), electrostatic terms (ELAC), and bonded terms (BLAC). In the following, we test the performance of the various correction schemes for three different systems: ethanol, sulfite oxidase, and haem oxidase. The results are described in three separate sections. For each system, we study six different approaches: the additive scheme (Add, i.e., without any link-atom corrections), the subtractive scheme with van der Waals (VLAC), electrostatic (ELAC), and bonded link-atom corrections (BLAC), the latter in two variants (BLAC1 or BLAC2 and BLAC2J) and mechanical embedding (ME, using a subtractive scheme and VLAC). ELAC and the three variants of BLAC always also include VLAC. Therefore, we use the abbreviation Sub for the subtractive scheme involving only VLAC (emphasizing that this is the standard approach for the subtractive scheme in ComQum). For ethanol, we tested a few additional combinations.

### Ethanol

We first tested all methods on a very simple model system, viz. ethanol, in which methanol was used as the QM system in QM/MM calculations and the results were compared to a QM calculation on the full ethanol molecule. Nine different calculations were run for this system: Add, Sub, two variants of ELAC, BLAC1, BLAC1J, BLAC2, BLAC2J, and ME, as well as ELAC combined with BLAC2J. The two variants of ELAC used the same ESP charges for the QM system. However, for the MM system, they used either the ESP charges of ethanol (ELAC2) or the ESP charges for methanol for all methanol atoms except HL, the ethanol ESP charges for the H21–H23 atoms on the terminal methyl group, whereas the charge on the CL atom was adapted to give a vanishing net charge (ELAC1). The latter approach is similar to what is used for the two enzyme systems, for which ESP charges for the full MM system are not available. The two sets of charges are shown in Table 2.

The results of the QM/MM calculations on ethanol are collected in Table 3. The first column gives the total QM/MM energies, relative to the Add calculations. This is only to illustrate that the QM/MM energy depends on the MM force field and therefore give different results for the various calculations.

**Table 3:** Results for the QM/MM calculations on ethanol.

	***E*_QM/MM_**	***E*_EtOH_**	**RMSD**	**MAD_bond_**	**MAD_angle_**	**MAD_dihed_**
Add	0.0	2.2	0.014	0.006	1.15	0.67
Sub	0.0	2.2	0.014	0.006	1.14	0.66
ELAC	−52.1	2.1	0.015	0.006	1.19	0.66
ELAC2	−5.1	2.0	**0.012**	0.006	1.07	0.74
BLAC1	−0.1	2.0	0.013	0.006	1.01	0.58
BLAC1J	0.3	**1.7**	0.013	0.006	1.02	0.58
BLAC2	−8.3	2.9	0.036	**0.001**	0.80	0.38
BLAC2J	−8.1	2.9	0.036	0.002	0.80	0.38
ELAC+BLAC2J	−60.2	3.3	0.039	0.002	**0.77**	**0.37**
ME	−56.5	**1.7**	0.016	0.005	1.12	0.49
MM		3.8	0.015	0.014	0.78	0.83

*E_QM/MM_ is the total QM/MM energy (kJ/mol), E_EtOH_ is the QM energy of the whole ethanol molecule (kJ/mol), RMSD is the RMS difference of the coordinates, MAD_bond_ (Å), MAD_angle_ (°), and MAD_dihed_ (°) are the mean absolute deviation compared to the ethanol molecule optimized by QM. The best value in each column is marked in bold face*.

The second column gives the QM energy of the QM/MM optimized structure of ethanol. It can be seen that BLAC1J and ME give the lowest energy, 1.7 kJ/mol above the QM minimum. On the other hand, ELAC+BLAC1 gives the highest energy, 3.3 kJ/mol. Thus, the variation in energies is small, showing that all structures give excellent structures. A MM minimisation with the GAFF force field gives a slightly higher energy, 3.8 kJ/mol (row MM in Table 3).

The third column shows the root-mean-squared deviation (RMSD) of the coordinates from the optimized QM structure of ethanol. ELAC2 gives the lowest RMSD (0.012 Å), followed by the two BLAC1 variants (0.013 Å), as well as Add, Sub and ME (0.014–0.016 Å). The three variants involving BLAC2 give slightly higher values, 0.036–0.039 Å.

Finally, the three last columns give the mean absolute deviation (MAD) for the 8 bonds, the 13 angles and the 12 dihedrals in the molecule. For the bonds, the three BLAC2 variants give minimal errors (0.001–0.002 Å), whereas the other methods give slightly higher errors, 0.005–0.006 Å. The same applies for the angles and the dihedrals, the three BLAC2 variants are still the best (0.8 and 0.4°), whereas the other methods give slightly larger errors, 0.8–1.2° and 0.5–0.7°.

In conclusion, the test calculations show that the BLAC approaches give the best result, but it depends on the force field used. The best structures are obtained with the Hess2FF force field, which is tailored for the molecule and the QM method. ELAC sometimes improves the results, sometimes not. Sub and Add give similar results and in the differences among the various method are minimal for this small test molecule.

### Sulfite oxidase

Next, we studied a more realistic enzyme system, viz. sulfite oxidase. Based on our recent QM-cluster (Van Severen et al., 2014) and QM/MM (Caldararu et al., 2018) studies, we considered the S → OMo mechanism, in which the S atom of the sulfite substrate attacks the equatorial oxo group of Mo^VI^, directly forming a Mo^IV^-sulfate intermediate (Im), via a first transition state TS1 (Figure 2). The sulfate product then dissociates into the second coordination sphere of the Mo ion via a second transition state (TS2).

For all five states, we tested six different methods: Add, Sub, ELAC, BLAC2, BLAC2J, and ME. For all methods, we compare the QM/MM results obtained with the small DMDT model of the molybdopterin ligand with those obtained with a standard (subtractive with VLAC) QM/MM calculation with the full MPT ligand (Figure 2). Initially, we tested also BLAC1, but it failed for all systems. The RS state with ME could be obtained only if the S_Sub_−O2 distance was fixed at 2.43 Å (taken from the reference structure).

Table 4 shows the RMSD deviations of the small QM system between the MPT and DMDT calculations. It can be seen that all calculations give similar results, with a RMSD of 0.02–0.10 Å. The RMSD is typically lowest for the Im and TS1 states.

**Table 4:** RMS deviations (Å) of the various QM systems for sulfite oxidase, compared to the QM/MM structures optimized with the full MPT ligand.

	**Add**	**Sub**	**ELAC**	**BLAC1**	**BLAC2J**	**ME**
RS	0.079	0.085	0.085	0.094	0.094	0.101
Ts1	0.025	0.027	0.028	0.040	0.041	0.053
Im	0.019	0.016	0.020	0.036	0.036	0.071
Ts2	0.063	0.070	0.074	0.083	0.083	0.087
PS	0.064	0.070	0.075	0.082	0.083	0.087
Average	0.047	0.050	0.052	0.063	0.064	0.078

The smallest RMSD is always found for Add and Sub methods, with an average RMSD of 0.05 Å for the five states. However, ELAC also gives a similar average, whereas that of the other three methods is somewhat larger, 0.06 and 0.08 Å for BLAC and ME, respectively. This increase in the RMSD is not caused by a single structure, but is seen for all structures.

In Table S1, the Mo–ligand and S_Sub_−O distances for all structures are listed. It can be seen that these key distances are well preserved in the truncated calculations. The best results are again obtained for Add and Sub, for which the average difference for the nine distances and five sets of structures is only 0.008 Å. The maximum deviation is 0.14 Å for Add and 0.16 Å for Sub, in both cases obtained for the non-bonded S_Sub_–O2 distance in the RS structure. Besides this distance, the largest deviation is only 0.02–0.03 Å. The results for the other four sets of calculations were slightly worse, with an average error of 0.017 Å for ME and 0.009 Å for the other three approaches. The maximum error is 0.14–0.17 Å, again for S_Sub_–O2 distance in the RS structure, except for ME (Mo–O2 distance of the Im state).

**Figure 4:**
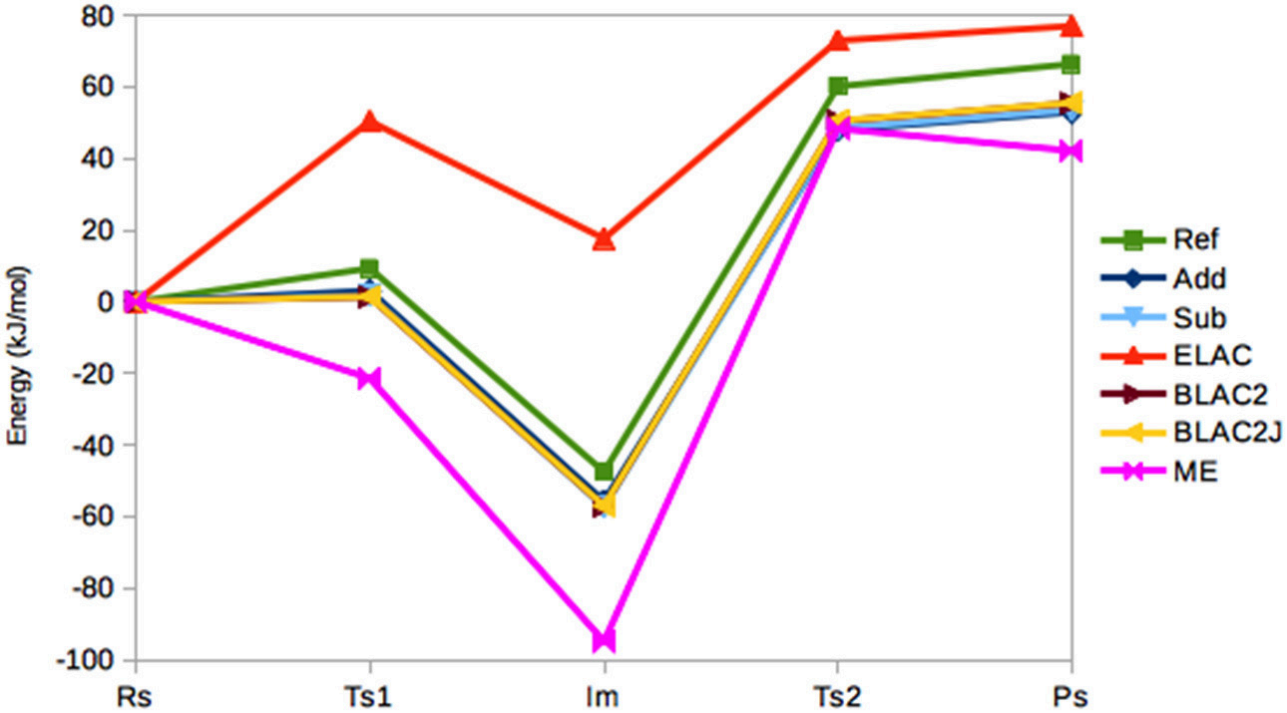
Relative energies (kJ/mol) of the five states in the sulfite oxidase reaction, obtained with the various QM/MM methods.

Figure 4 shows the energies (relative to the RS state) in the seven sets of calculations. It can be seen that the Add, Sub, BLAC2, and BLAC2J methods give very similar results. In fact, the two sets of BLAC2 energies differ by only 0.1 kJ/mol for all states and these methods differ by 0–2 kJ/mol from Sub, and slightly more from Add (2 kJ/mol on average). The Add and Sub results agree within 2 kJ/mol. All these curves follow quite closely the reference with a systematic underestimation that increases from 6–8 kJ/mol for TS1 to 11–13 kJ/mol for PS. On average, all methods give a MAD of 10 kJ/mol, lowest for BLAC2J.

On the other hand, ME and ELAC give appreciably larger errors, with MADs of 29 and 32 kJ/mol, respectively and maximum errors of 47 and 65 kJ/mol. For ME, the problem is related to the failure to find the RS state—different restraints for this state may translate the curve upwards and therefore reduce the error, but it always remains worse than the other four methods.

Thus, we can conclude that all methods give reasonable structures for sulfite oxidase, although ME has problem with one of the states. However, ME and ELAC give quite large errors for the energies. In general, Add and Sub seem to give the best (and similar) results.

### Haem oxygenase

The third test case is haem oxygenase, for which we studied the conversion of oxophlorin (OXF) to verdohaem (Alavi et al., 2017). As is shown in Figure 3, this involves five states and four transition states. It starts from the Fe^III^-OXF–${\text{O}}_{2}^{-}$ complex in the doublet state (**1**), which has one unpaired electron on each of the three moieties, in analogy with previous studies (Alavi et al., 2017; Gheidi et al., 2017). In the first step of the reaction, the terminal oxygen atom in O_2_ (O2) reacts with the C4B atom of OXF, forming a bridging intermediate (**2**). In the next step, the O–O bond is cleaved, giving intermediate **3**. Next, the O2 atom reacts with another atom in the OXF ring (C1C), forming a four-membered ring in intermediate **4**. Finally, the C4B–CMC and C1C–CMC bonds are cleaved, and CO dissociates, giving rise to verdohaem (**5**). These states are separated by four transition states (**T1–T4**). We study the effect of moving the side chains of the OXF ring from the QM to the MM system.

This test case is challenging for at least two reasons. First, the electronic structure is more complicated than for the other two test cases, with several antiferromagnetically coupled open-shell moieties. Second, the OXF ring, for which we test the effect of truncation, is involved in the reaction. In fact, the C1C and C4B atoms are both only two bonds away from the HL atoms in the truncated OXT model.

As for sulfite oxidase, we tested six different methods with the truncated OXT model, Add, Sub, ELAC, BLAC1, BLAC2J, and ME. In contrast to sulfite oxidase, the simplest BLAC1 approach, with standard GAFF parameters for both OXF and OXT worked well. Considering the similar results for BLAC2 and BLAC2J for sulfite oxidase, we did not test BLAC2.

As for sulfite oxidase, we used the QM/MM calculations (subtractive with VLAC) with the full OXF ligand as the reference and study how the various QM/MM calculations with OXT reproduce these calculations in terms of the RMSD deviation for the entire (truncated) QM system, key distances and energies. The RMSD deviations of the nine different systems are shown in Table 5. It can be seen that most methods give similar results with an average RMSD of 0.06 Å. Add gives the lowest RMSD for most systems, but that of Sub is very similar and sometimes lower. The largest RMSD (0.09–0.10 Å) is typically found for state **3**, for which ME actually gives the best results and the latter method also gives the lowest maximum RMSD.

**Table 5:** RMS deviations (Å) of the various QM systems of haem oxygenase, compared to the QM/MM structures optimized with the full OXF ligand.

	**Add**	**Sub**	**ELAC**	**BLAC1**	**BLAC2J**	**ME**
1	0.053	0.066	0.072	0.071	0.058	0.073
T1	0.046	0.053	0.057	0.057	0.069	0.057
2	0.042	0.050	0.053	0.055	0.065	0.053
T2	0.047	0.052	0.054	0.058	0.064	0.056
3	0.096	0.098	0.099	0.106	0.110	0.091
T3	0.082	0.083	0.087	0.089	0.126	0.083
4	0.086	0.086	0.091	0.092	0.104	0.094
T4	0.030	0.031	0.035	0.043	0.069	0.037
5	0.032	0.031	0.033	0.043		0.034
Average	0.057	0.061	0.065	0.068	0.083	0.064

However, for the BLAC1 method, the RMSD is slightly larger, with an average of 0.07 Å and a maximum of 0.11 Å (still for **3**). For BLAC2J, the results are even worse (0.08 Å on average and a maximum of 0.13 Å for **T3**). In particular, BLAC2J failed to converge to any reasonable structure for the product (**5**). This most likely reflects that the force fields, especially that of BLAC2J, were determined for the starting structure **1** (note that BLAC2J gives the second-best structure for that state) and was then used unchanged for the other states. This is clearly suboptimal for structures later in the reaction mechanism. Of course, we could have determined new force fields for each intermediate and transition state in the mechanism, but this would have required much large computational and manual effort. Moreover, it would have given problems in the calculated energies, because the force field would be different for every state, making the energies not comparable. Thus, we do not recommend the BLAC approaches if the reaction is within three bonds of the XL atoms.

These trends in the RMSD are reflected also in the individual distances in the complexes. In Table S2, we examine the six Fe–N/O distances, as well as distances involving the reacting O2, C1C, C4B, and CMC atoms. It can be seen that all methods give similar MAD and maximum deviations from the reference distances (0.02–0.03 and 0.29–0.32 Å, respectively). However, Add and Sub still give the best results on average and BLAC2J the worst. All methods give large errors for the Fe–N_His_ distances in the **3, T4** and **4** states (0.26–0.32 Å too short). All methods also give a large error for the O2–C1C distance in state **3** (0.18–0.27 Å too short).

**Figure 5:**
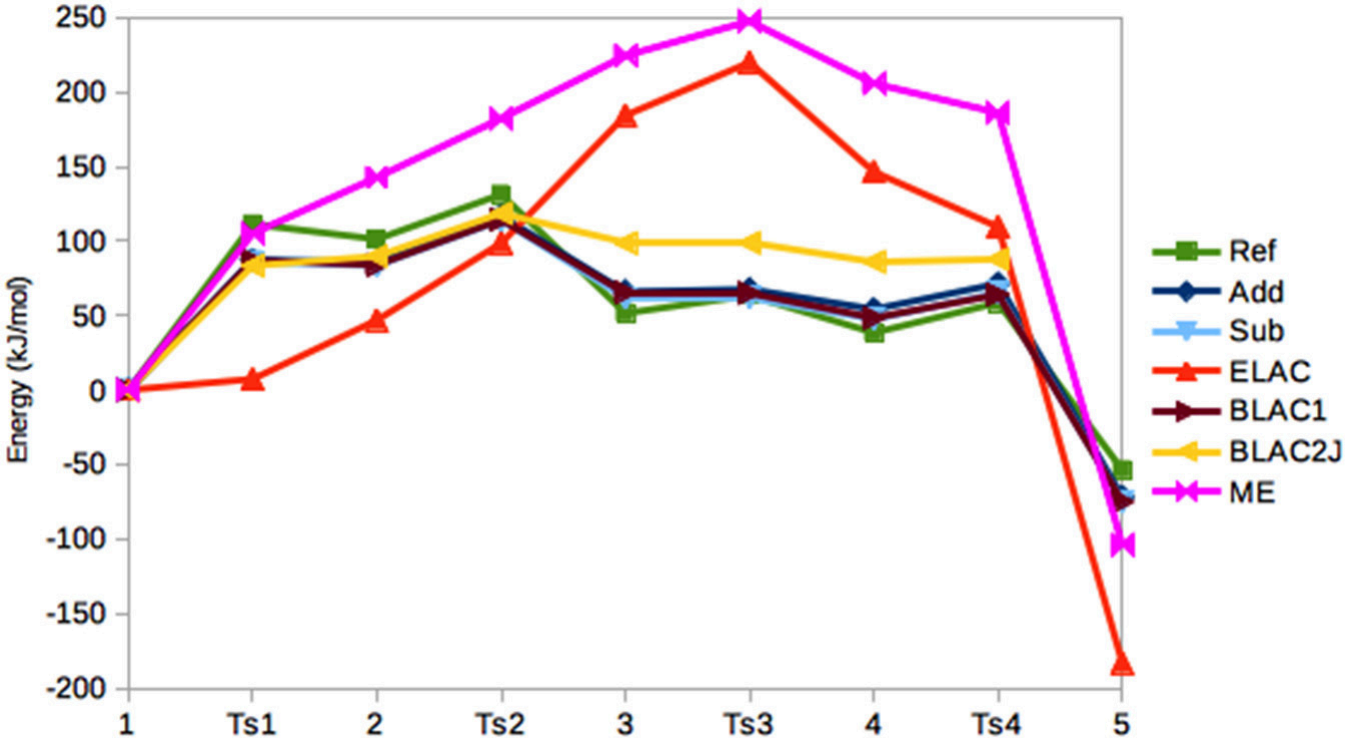
Relative energies (kJ/mol) of the five states in the haem oxygenase reaction, obtained with the various QM/MM methods.

In Figure 5, the relative energies of the various states are shown. It can be seen that the Add and Sub methods still give similar results, with a MAD of only 4 kJ/mol. However, the Sub and BLAC1 methods give even more similar results with a MAD of only 1.4 kJ/mol. This reflects that GAFF parameters for OXT and OXF differ only for a few bonds, angles, and dihedrals around the periphery of the ring, which apparently do not affect the results much. These three methods also reproduce the reference calculations fairly well, with MADs of 14–15 kJ/mol, with Sub giving the smallest error. The maximum error, 24–25 kJ/mol, is obtained for **T2**.

BLAC2J gives somewhat worse energies (MAD = 30 kJ/mol, with a maximum error of 47 kJ/mol for both **3** and **4**), especially for the later states in the reaction, reflecting that the force field is worse for these states. As for sulfite oxidase, the results for ELAC and ME are much worse, with MADs of 96–100 kJ/mol and maximum errors of 156–184 kJ/mol.

Finally, we checked also the electronic structures in the various calculations. However, they showed only a small variation between the various methods. For example, for the spin densities on Fe (shown in Table S3), Add, Sub, BLAC1 and BLAC2J give the same MAD from the reference calculations, 0.06 *e*, and the MADs of ELAC and ME are only slightly larger, 0.07 and 0.08 *e*, respectively. The spin density is lower for all calculations with OXT, except for the **1** state and the difference is largest for **3** and **T3** (0.09–0.12 *e*).

In conclusion, Sub and Add again give the best results, but those of BLAC1 are also good. Again, ELAC and ME give large errors in the energies and BLAC2J has problems with geometries of the later states in the reaction.

## Conclusions

In this paper, we have tried to clarify similarities and differences between the subtractive and additive QM/MM schemes. In our view, the primary difference is that the subtractive scheme allows for an attempt to correct for errors introduced by the link atoms. This correction can be introduced for three different types of interactions: van der Waals, electrostatic, and bonded interactions. Different software implements different corrections and it is also partly up to the user to define the level of correction (when setting up the force field for the QM system). For example, our ComQum software automatically implements the van der Waals correction, whereas ONIOM with electrostatic embedding implements also the electrostatic correction. Both software can implement the bonded correction, but typically do not do so.

Of course, the corrections come at some extra cost. For the van der Waals correction (VLAC), the cost is minimal: It requires van der Waals parameters of the HL atoms, which can almost always be taken from standard parameters for hydrogen atoms in the used MM force field (using the rules for atom types of the force field). Beside these parameters, the calculations can be automatically set up from a prmtop file for the full system with no extra effort, as in the ComQum implementation.

For the electrostatic link-atom corrections (ELAC), QM charges of the QM region are required, both with HL and CL atoms. This can be obtained from most QM software at a small extra cost and be automatized. Moreover, such charges are normally already available, because most QM/MM studies start with a MD equilibration of the full system (including solvent), for which a proper charge model of the QM system is needed. However, the charges on the CL atoms are ambiguous and ESP charges of buried atoms are poorly defined, which becomes a serious problem for large QM regions. Therefore, we have not seen any advantage for the electrostatic correction, at least not when the link atoms are rather close to the reactive atoms (Hu et al., 2011b). Moreover, the test calculations in this article indicate that ELAC can give rise to problems with energies in a reaction sequence. ELAC is implemented in the ONIOM software, but in practice it gives often severe convergence problems and is therefore seldom used. Instead, alternative approaches have been implemented, based on iterative calculations with mechanical embedding and updated charges (Kawatsu et al., 2011; Dutta and Mishra, 2014; Wójcik et al., 2014). It could therefore be recommended that ONIOM implements electrostatic embedding with only VLAC, which is a very stable approach in ComQum.

For the bonded link-atom correction (BLAC), an accurate MM force field for the QM region is required. Nowadays, the general MM force field for organic and drug-like molecules often provide the required parameters. However, it is unclear whether these (together with the MM parameters of the full system) are accurate enough to give any advantage of this approach. The alternative is to make a tailored force field for the QM region, both when truncated and in the full protein. In this article, we have tested both approaches. For the simple ethanol test case, the best results were actually obtained with BLAC2, i.e., with the optimized Hess2FF force field. However, for the two enzyme systems, the results were worse, especially if the reactive site is close to the link atoms. Therefore, we cannot recommend BLAC for general use.

Beside the parameters needed for these link-atom corrections, there is no difference in the requirement of MM parameters for the subtractive and additive QM/MM schemes; without any link-atom corrections, the two schemes should give identical results if correctly implemented and require exactly the same MM parameters. However, in practice there may be differences because the subtractive scheme is typically based on a standard (general-purpose) MM software, whereas the additive scheme is based on a software tailored for QM/MM. In particular, most MM software refuse to run if any MM parameters are missing. Therefore, subtractive calculations normally require a full set of MM parameters, also for the QM region. However, these can be dummy (zeroed) parameters, because they cancel in the QM/MM calculations. Moreover, also the additive scheme requires these parameters for an initial MD equilibration of the full solvated system. The same parameters can normally be used throughout a reaction mechanism (again because these MM terms cancel in Equation 4).

Thus, our conclusion is that intrinsically, the subtractive and additive QM/MM schemes are equivalent if properly implemented. The subtractive scheme allows the introduction of various link-atom corrections, at the expense of requiring more MM parameters. For van der Waals and electrostatic corrections, the extra cost is minimal and fully automatic, whereas for the bonded corrections, significant extra effort may be needed. Of course, the same corrections may be implemented also in an additive scheme, by picking proper terms, but this goes outside a standard implementation (and also a strict definition) of the additive scheme and therefore should be thoroughly specified.

In practice, the subtractive scheme is easier to implement and maintain (standard QM and MM software are used). On the other hand, the additive scheme may be somewhat easier to set up and can be tailored for QM/MM calculations. Moreover, if (a major part of) the MM system is fixed, calculations can be somewhat sped up by not calculating the MM energy and forces for the fixed atoms. In our test calculations, the additive and subtractive calculations with VLAC, but no other corrections (i.e., Add and Sub) give closely similar results, showing that the VLAC has only a minor influence on the results. Thus, both approaches can be recommended for QM/MM calculations.

We have also included mechanical embedding (within a subtractive scheme with VLAC) in the comparison. However, this approach gave rather poor structures, especially for sulfite oxidase. Moreover, the energies were quite poor, although this may be partly attributed to the fact that the reference energies employed electrostatic embedding and not mechanical embedding. For a more fair comparison, reference energies obtained with very large QM systems should be used, as in our previous study (Hu et al., 2011b).

Finally, we want to emphasize the importance of specifying exactly what is done in the QM/MM calculations, owing to the many different implementations. Obviously, it is not enough to say that a subtractive or additive scheme is used. Instead, for a subtractive scheme, it must be specified what type of link-atom corrections is applied (van der Waals, electrostatic, or bonded). In addition, the treatment of QM–MM electrostatics (mechanical or electrostatic embedding) must be specified, together with a detailed account of what charges are included in the point-charge model, if any charge redistribution scheme is employed and how charges on CL atoms are obtained. Finally, the treatment of link atoms need to be specified, as well as the relation between the coordinates of the HL and CL atoms.

## Author contributions

LC: Performed most of the calculations; UR: Did some calculations, designed the project, and wrote the article.

### Conflict of interest statement

The authors declare that the research was conducted in the absence of any commercial or financial relationships that could be construed as a potential conflict of interest.
