# Supplementary material for: On the Difference Between Additive and Subtractive QM/MM Calculations
Source: Front Chem. 2018 Apr 3;6:89. doi: 10.3389/fchem.2018.00089 (PMC5891596; doi:10.3389/fchem.2018.00089)
Supplement: Supplementary file 1 [file Table1.PDF]

*Supplementary material*

# On the difference between additive and subtractive QM/MM calculations

**Lili Cao & Ulf Ryde ***

Department of Theoretical Chemistry, Lund University, Chemical Centre, P. O. Box 124, SE-221 00 Lund, Sweden

Correspondence to Ulf Ryde, E-mail: Ulf.Ryde@teokem.lu.se,

Tel: +46 – 46 2224502, Fax: +46 – 46 2228648

**2018-03-02**

**Table S1.** Mo–ligand and $S_{Sub}$–O distances (Å) for the various QM/MM approaches for sulfite oxidase. The last two rows show the MAD and maximum difference from the QM/MM reference calculation with MPT. Atom names are defined in Figure 1.

| Bond | State | Ref | Add | Sub | ELAC | BLAC2 | BLAC2J | ME |
|---|---|---|---|---|---|---|---|---|
| Mo–$S_{Cys}$ | RS | 2.43 | 2.43 | 2.43 | 2.43 | 2.42 | 2.42 | 2.44 |
| | TS1 | 2.43 | 2.43 | 2.43 | 2.43 | 2.42 | 2.42 | 2.43 |
| | Im | 2.43 | 2.43 | 2.43 | 2.43 | 2.42 | 2.42 | 2.42 |
| | TS2 | 2.41 | 2.41 | 2.41 | 2.41 | 2.41 | 2.41 | 2.40 |
| | PS | 2.41 | 2.41 | 2.41 | 2.41 | 2.41 | 2.41 | 2.41 |
| Mo–S1 | RS | 2.50 | 2.47 | 2.47 | 2.47 | 2.46 | 2.46 | 2.46 |
| | TS1 | 2.48 | 2.46 | 2.46 | 2.46 | 2.45 | 2.45 | 2.45 |
| | Im | 2.41 | 2.40 | 2.40 | 2.40 | 2.40 | 2.40 | 2.42 |
| | TS2 | 2.33 | 2.32 | 2.32 | 2.32 | 2.32 | 2.32 | 2.32 |
| | PS | 2.33 | 2.32 | 2.32 | 2.32 | 2.32 | 2.32 | 2.32 |
| Mo–S2 | RS | 2.47 | 2.45 | 2.45 | 2.45 | 2.45 | 2.45 | 2.47 |
| | TS1 | 2.46 | 2.45 | 2.45 | 2.45 | 2.45 | 2.45 | 2.46 |
| | Im | 2.43 | 2.43 | 2.43 | 2.42 | 2.43 | 2.43 | 2.45 |
| | TS2 | 2.39 | 2.38 | 2.38 | 2.38 | 2.39 | 2.39 | 2.38 |
| | PS | 2.39 | 2.38 | 2.38 | 2.38 | 2.39 | 2.39 | 2.38 |
| Mo–O1 | RS | 1.75 | 1.75 | 1.75 | 1.75 | 1.75 | 1.75 | 1.74 |
| | TS1 | 1.74 | 1.74 | 1.74 | 1.74 | 1.74 | 1.74 | 1.73 |
| | Im | 1.73 | 1.73 | 1.73 | 1.73 | 1.73 | 1.73 | 1.72 |
| | TS2 | 1.73 | 1.73 | 1.73 | 1.73 | 1.74 | 1.74 | 1.74 |
| | PS | 1.73 | 1.73 | 1.73 | 1.73 | 1.74 | 1.74 | 1.73 |
| Mo–O2 | RS | 1.81 | 1.84 | 1.84 | 1.84 | 1.84 | 1.84 | 1.85 |
| | TS1 | 1.96 | 1.95 | 1.95 | 1.95 | 1.95 | 1.95 | 1.99 |
| | Im | 2.26 | 2.28 | 2.28 | 2.28 | 2.27 | 2.27 | 2.12 |
| | TS2 | 3.70 | 3.70 | 3.70 | 3.70 | 3.70 | 3.70 | 3.70 |
| | PS | 3.85 | 3.85 | 3.85 | 3.85 | 3.85 | 3.85 | 3.85 |
| $S_{Sub}$–O3 | RS | 1.56 | 1.56 | 1.55 | 1.55 | 1.55 | 1.55 | 1.52 |
| | TS1 | 1.54 | 1.54 | 1.54 | 1.54 | 1.54 | 1.54 | 1.50 |
| | Im | 1.53 | 1.53 | 1.53 | 1.53 | 1.53 | 1.53 | 1.51 |
| | TS2 | 1.54 | 1.54 | 1.54 | 1.54 | 1.54 | 1.54 | 1.51 |
| | PS | 1.54 | 1.54 | 1.54 | 1.54 | 1.54 | 1.54 | 1.51 |
| $S_{Sub}$–O4 | RS | 1.53 | 1.53 | 1.53 | 1.53 | 1.53 | 1.53 | 1.51 |
| | TS1 | 1.52 | 1.52 | 1.52 | 1.52 | 1.52 | 1.52 | 1.49 |
| | Im | 1.50 | 1.50 | 1.50 | 1.50 | 1.50 | 1.50 | 1.50 |
| | TS2 | 1.51 | 1.51 | 1.51 | 1.51 | 1.51 | 1.51 | 1.50 |
| | PS | 1.51 | 1.51 | 1.51 | 1.51 | 1.51 | 1.51 | 1.50 |
| $S_{Sub}$–O5 | RS | 1.54 | 1.54 | 1.54 | 1.54 | 1.54 | 1.54 | 1.51 |
| | TS1 | 1.52 | 1.52 | 1.52 | 1.52 | 1.52 | 1.52 | 1.50 |
| | Im | 1.51 | 1.51 | 1.51 | 1.51 | 1.51 | 1.51 | 1.50 |
| | TS2 | 1.52 | 1.52 | 1.52 | 1.52 | 1.52 | 1.52 | 1.51 |
| | PS | 1.52 | 1.52 | 1.52 | 1.52 | 1.52 | 1.52 | 1.52 |
| $S_{Sub}$–O2 | RS | 2.43 | 2.29 | 2.27 | 2.28 | 2.26 | 2.26 | 2.42 |
| | TS1 | 2.00 | 2.00 | 2.00 | 2.00 | 2.00 | 2.00 | 1.99 |
| | Im | 1.59 | 1.58 | 1.58 | 1.58 | 1.58 | 1.58 | 1.64 |
| | TS2 | 1.53 | 1.53 | 1.53 | 1.53 | 1.53 | 1.53 | 1.54 |
| | PS | 1.53 | 1.53 | 1.53 | 1.53 | 1.53 | 1.53 | 1.54 |
| MAD | | | 0.008 | 0.008 | 0.009 | 0.009 | 0.009 | 0.017 |
| Max | | | 0.14 | 0.16 | 0.15 | 0.17 | 0.17 | 0.14 |

**Table S2.** Key distances (Å) for the various QM/MM approaches for haem oxygenase. The last two rows show the MAD and maximum difference from the reference QM/MM calculation with OXF. Atom names are defined in Figure 2.

| Bond | State | Med | Add | Sub | ELAC | BLAC1 | BLAC2J | ME |
|---|---|---|---|---|---|---|---|---|
| Fe–$N_{His}$ | 1 | 2.04 | 2.05 | 2.05 | 2.02 | 2.05 | 2.05 | 2.04 |
| | Ts1 | 2.01 | 2.00 | 2.00 | 1.99 | 2.01 | 2.01 | 2.00 |
| | 2 | 2.05 | 2.04 | 2.04 | 2.03 | 2.04 | 2.05 | 2.03 |
| | Ts2 | 2.43 | 2.37 | 2.37 | 2.37 | 2.37 | 2.39 | 2.36 |
| | 3 | 2.41 | 2.15 | 2.15 | 2.13 | 2.15 | 2.15 | 2.14 |
| | Ts3 | 2.46 | 2.17 | 2.17 | 2.14 | 2.18 | 2.16 | 2.16 |
| | 4 | 2.50 | 2.20 | 2.20 | 2.17 | 2.21 | 2.19 | 2.19 |
| | Ts4 | 2.49 | 2.46 | 2.46 | 2.47 | 2.46 | 2.47 | 2.46 |
| | 5 | 2.45 | 2.42 | 2.42 | 2.42 | 2.42 | | 2.42 |
| Fe–N1 | 1 | 2.00 | 2.01 | 2.01 | 2.01 | 2.01 | 2.00 | 2.02 |
| | Ts1 | 2.01 | 2.02 | 2.02 | 2.02 | 2.03 | 2.02 | 2.02 |
| | 2 | 2.00 | 2.01 | 2.02 | 2.01 | 2.02 | 2.01 | 2.01 |
| | Ts2 | 2.00 | 2.00 | 2.00 | 2.00 | 2.00 | 2.00 | 1.99 |
| | 3 | 2.00 | 2.01 | 2.01 | 2.01 | 2.02 | 2.00 | 2.01 |
| | Ts3 | 1.99 | 2.00 | 2.00 | 2.01 | 2.01 | 1.99 | 2.01 |
| | 4 | 1.99 | 2.00 | 2.00 | 2.00 | 2.00 | 1.99 | 2.00 |
| | Ts4 | 1.99 | 2.01 | 2.01 | 2.01 | 2.01 | 2.00 | 2.01 |
| | 5 | 1.98 | 1.99 | 1.99 | 1.99 | 2.00 | | 1.99 |
| Fe–N2 | 1 | 2.02 | 2.02 | 2.02 | 2.02 | 2.03 | 2.03 | 2.03 |
| | Ts1 | 1.97 | 1.97 | 1.97 | 1.97 | 1.98 | 1.97 | 1.97 |
| | 2 | 1.93 | 1.93 | 1.93 | 1.93 | 1.94 | 1.93 | 1.93 |
| | Ts2 | 1.93 | 1.92 | 1.92 | 1.92 | 1.93 | 1.92 | 1.92 |
| | 3 | 2.00 | 1.99 | 1.99 | 1.99 | 2.00 | 1.99 | 2.00 |
| | Ts3 | 2.00 | 1.99 | 1.99 | 1.99 | 2.00 | 2.00 | 2.00 |
| | 4 | 1.99 | 1.98 | 1.98 | 1.98 | 1.99 | 1.99 | 1.99 |
| | Ts4 | 2.00 | 2.00 | 2.00 | 2.00 | 2.01 | 2.01 | 2.00 |
| | 5 | 2.05 | 2.05 | 2.05 | 2.06 | 2.06 | | 2.06 |
| Fe–N3 | 1 | 2.01 | 2.01 | 2.01 | 1.99 | 2.01 | 2.02 | 2.00 |
| | Ts1 | 2.02 | 2.03 | 2.03 | 2.03 | 2.03 | 2.03 | 2.03 |
| | 2 | 2.04 | 2.05 | 2.05 | 2.04 | 2.05 | 2.05 | 2.05 |
| | Ts2 | 2.08 | 2.13 | 2.13 | 2.12 | 2.12 | 2.11 | 2.13 |
| | 3 | 2.05 | 2.02 | 2.02 | 2.00 | 2.02 | 2.03 | 2.01 |
| | Ts3 | 2.01 | 2.00 | 2.00 | 1.98 | 2.00 | 2.01 | 1.98 |
| | 4 | 1.96 | 1.96 | 1.96 | 1.95 | 1.96 | 1.97 | 1.95 |
| | Ts4 | 1.97 | 1.98 | 1.98 | 1.98 | 1.98 | 1.99 | 1.98 |
| | 5 | 2.01 | 2.02 | 2.02 | 2.01 | 2.02 | | 2.02 |
| Fe–N4 | 1 | 2.01 | 2.02 | 2.02 | 2.01 | 2.02 | 2.02 | 2.01 |
| | Ts1 | 2.04 | 2.05 | 2.05 | 2.05 | 2.05 | 2.05 | 2.05 |
| | 2 | 2.06 | 2.07 | 2.07 | 2.07 | 2.08 | 2.07 | 2.07 |
| | Ts2 | 2.10 | 2.14 | 2.14 | 2.14 | 2.14 | 2.14 | 2.15 |
| | 3 | 2.04 | 2.03 | 2.03 | 2.01 | 2.02 | 2.02 | 2.02 |
| | Ts3 | 2.02 | 2.01 | 2.01 | 2.00 | 2.01 | 2.01 | 2.00 |
| | 4 | 2.01 | 2.00 | 2.00 | 1.99 | 2.00 | 2.00 | 1.99 |
| | Ts4 | 2.01 | 2.02 | 2.02 | 2.02 | 2.02 | 2.02 | 2.02 |
| | 5 | 1.99 | 2.01 | 2.01 | 2.01 | 2.01 | | 2.01 |

| | | | | | | | | |
|---|---|---|---|---|---|---|---|---|
| Fe–O1 | 1 | 1.82 | 1.82 | 1.82 | 1.84 | 1.82 | 1.82 | 1.83 |
| | Ts1 | 1.88 | 1.90 | 1.90 | 1.91 | 1.90 | 1.90 | 1.90 |
| | 2 | 1.81 | 1.83 | 1.83 | 1.85 | 1.83 | 1.84 | 1.84 |
| | Ts2 | 1.75 | 1.75 | 1.75 | 1.76 | 1.75 | 1.75 | 1.75 |
| | 3 | 1.63 | 1.64 | 1.64 | 1.65 | 1.64 | 1.64 | 1.65 |
| | Ts3 | 1.63 | 1.64 | 1.64 | 1.65 | 1.64 | 1.64 | 1.65 |
| | 4 | 1.63 | 1.64 | 1.64 | 1.66 | 1.64 | 1.64 | 1.65 |
| | Ts4 | 1.63 | 1.63 | 1.63 | 1.64 | 1.63 | 1.63 | 1.63 |
| | 5 | 1.63 | 1.63 | 1.63 | 1.63 | 1.63 | | 1.63 |
| Fe–O2 | 1 | 2.66 | 2.66 | 2.66 | 2.68 | 2.66 | 2.66 | 2.68 |
| | Ts1 | 2.75 | 2.75 | 2.75 | 2.76 | 2.75 | 2.77 | 2.75 |
| | 2 | 2.76 | 2.74 | 2.74 | 2.74 | 2.74 | 2.76 | 2.74 |
| | Ts2 | 2.78 | 2.77 | 2.77 | 2.78 | 2.78 | 2.78 | 2.77 |
| | 3 | 3.51 | 3.46 | 3.45 | 3.45 | 3.44 | 3.40 | 3.46 |
| | Ts3 | 3.41 | 3.40 | 3.40 | 3.39 | 3.38 | 3.34 | 3.38 |
| | 4 | 3.27 | 3.26 | 3.25 | 3.24 | 3.25 | 3.23 | 3.23 |
| | Ts4 | 3.28 | 3.27 | 3.26 | 3.25 | 3.26 | 3.24 | 3.25 |
| | 5 | 3.42 | 3.41 | 3.40 | 3.40 | 3.41 | | 3.40 |
| O2–C4B | 1 | 3.47 | 3.49 | 3.50 | 3.52 | 3.50 | 3.44 | 3.48 |
| | Ts1 | 1.80 | 1.80 | 1.80 | 1.80 | 1.80 | 1.80 | 1.80 |
| | 2 | 1.49 | 1.52 | 1.52 | 1.54 | 1.53 | 1.56 | 1.54 |
| | Ts2 | 1.45 | 1.46 | 1.46 | 1.47 | 1.46 | 1.47 | 1.47 |
| | 3 | 1.36 | 1.38 | 1.38 | 1.39 | 1.39 | 1.39 | 1.38 |
| | Ts3 | 1.40 | 1.41 | 1.41 | 1.41 | 1.41 | 1.41 | 1.41 |
| | 4 | 1.47 | 1.47 | 1.47 | 1.47 | 1.47 | 1.45 | 1.48 |
| | Ts4 | 1.44 | 1.43 | 1.43 | 1.44 | 1.44 | 1.43 | 1.44 |
| | 5 | 1.35 | 1.35 | 1.35 | 1.35 | 1.36 | | 1.35 |
| O2–C1C | 1 | 3.34 | 3.34 | 3.35 | 3.38 | 3.36 | 3.30 | 3.36 |
| | Ts1 | 2.77 | 2.75 | 2.75 | 2.74 | 2.75 | 2.75 | 2.75 |
| | 2 | 2.80 | 2.77 | 2.76 | 2.76 | 2.76 | 2.77 | 2.77 |
| | Ts2 | 2.81 | 2.77 | 2.76 | 2.76 | 2.76 | 2.77 | 2.77 |
| | 3 | 2.40 | 2.17 | 2.17 | 2.18 | 2.12 | 2.21 | 2.22 |
| | Ts3 | 1.90 | 1.90 | 1.90 | 1.90 | 1.90 | 1.90 | 1.90 |
| | 4 | 1.46 | 1.45 | 1.45 | 1.45 | 1.44 | 1.44 | 1.45 |
| | Ts4 | 1.43 | 1.43 | 1.43 | 1.43 | 1.42 | 1.42 | 1.43 |
| | 5 | 1.35 | 1.35 | 1.35 | 1.35 | 1.34 | | 1.35 |
| O1–O2 | 1 | 1.28 | 1.27 | 1.27 | 1.27 | 1.27 | 1.28 | 1.27 |
| | Ts1 | 1.37 | 1.35 | 1.35 | 1.35 | 1.35 | 1.35 | 1.35 |
| | 2 | 1.52 | 1.47 | 1.48 | 1.46 | 1.47 | 1.45 | 1.46 |
| | Ts2 | 1.60 | 1.60 | 1.60 | 1.60 | 1.60 | 1.60 | 1.60 |
| | 3 | 3.38 | 3.33 | 3.32 | 3.31 | 3.31 | 3.21 | 3.29 |
| | Ts3 | 3.40 | 3.34 | 3.33 | 3.32 | 3.29 | 3.22 | 3.29 |
| | 4 | 3.37 | 3.29 | 3.28 | 3.26 | 3.26 | 3.23 | 3.23 |
| | Ts4 | 3.43 | 3.40 | 3.39 | 3.39 | 3.38 | 3.33 | 3.37 |
| | 5 | 3.78 | 3.76 | 3.75 | 3.74 | 3.74 | | 3.73 |
| C4B–CMC | 1 | 1.46 | 1.47 | 1.47 | 1.47 | 1.48 | 1.48 | 1.47 |
| | Ts1 | 1.51 | 1.51 | 1.51 | 1.52 | 1.52 | 1.52 | 1.52 |
| | 2 | 1.53 | 1.53 | 1.53 | 1.54 | 1.54 | 1.55 | 1.54 |
| | Ts2 | 1.54 | 1.54 | 1.54 | 1.54 | 1.54 | 1.57 | 1.54 |
| | 3 | 1.53 | 1.54 | 1.54 | 1.53 | 1.54 | 1.57 | 1.53 |
| | Ts3 | 1.54 | 1.54 | 1.54 | 1.54 | 1.54 | 1.57 | 1.54 |
| | 4 | 1.56 | 1.57 | 1.57 | 1.56 | 1.57 | 1.64 | 1.56 |
| | Ts4 | 1.79 | 1.79 | 1.79 | 1.79 | 1.79 | 1.80 | 1.79 |
| | 5 | 3.54 | 3.58 | 3.57 | 3.55 | 3.57 | | 3.55 |

| | | | | | | | | |
|---|---|---|---|---|---|---|---|---|
| CMC–O3 | 1 | 1.26 | 1.24 | 1.24 | 1.25 | 1.24 | 1.26 | 1.24 |
| | Ts1 | 1.24 | 1.23 | 1.23 | 1.23 | 1.23 | 1.24 | 1.23 |
| | 2 | 1.23 | 1.23 | 1.23 | 1.23 | 1.23 | 1.24 | 1.23 |
| | Ts2 | 1.23 | 1.22 | 1.22 | 1.23 | 1.22 | 1.23 | 1.23 |
| | 3 | 1.23 | 1.21 | 1.21 | 1.22 | 1.21 | 1.22 | 1.22 |
| | Ts3 | 1.21 | 1.21 | 1.21 | 1.21 | 1.21 | 1.22 | 1.21 |
| | 4 | 1.20 | 1.19 | 1.19 | 1.20 | 1.19 | 1.19 | 1.20 |
| | Ts4 | 1.19 | 1.18 | 1.18 | 1.18 | 1.18 | 1.19 | 1.18 |
| | 5 | 1.15 | 1.14 | 1.14 | 1.14 | 1.14 | | 1.15 |
| CMC–C1C | 1 | 1.46 | 1.47 | 1.47 | 1.47 | 1.47 | 1.47 | 1.47 |
| | Ts1 | 1.45 | 1.46 | 1.46 | 1.46 | 1.46 | 1.45 | 1.46 |
| | 2 | 1.45 | 1.46 | 1.46 | 1.46 | 1.46 | 1.45 | 1.46 |
| | Ts2 | 1.46 | 1.47 | 1.47 | 1.47 | 1.47 | 1.46 | 1.47 |
| | 3 | 1.49 | 1.51 | 1.51 | 1.51 | 1.51 | 1.49 | 1.51 |
| | Ts3 | 1.52 | 1.53 | 1.53 | 1.53 | 1.52 | 1.51 | 1.53 |
| | 4 | 1.56 | 1.57 | 1.57 | 1.57 | 1.57 | 1.57 | 1.57 |
| | Ts4 | 1.57 | 1.59 | 1.59 | 1.59 | 1.59 | 1.59 | 1.58 |
| | 5 | 2.65 | 2.73 | 2.72 | 2.72 | 2.73 | | 2.67 |
| | MAD | | 0.023 | 0.024 | 0.027 | 0.026 | 0.030 | 0.026 |
| | Max | | 0.30 | 0.30 | 0.32 | 0.29 | 0.30 | 0.31 |

**Table S3.** Spin densities on Fe (*e*) for the various QM/MM approaches for haem oxygenase. The last row shows the MAD difference from the reference QM/MM calculation with OXF.

| | Ref | Add | Sub | ELAC | BLAC1 | BLAC2J | ME |
|---|---|---|---|---|---|---|---|
| 1 | 0.93 | 0.97 | 0.97 | 0.99 | 0.98 | 0.96 | 0.99 |
| Ts1 | 0.65 | 0.60 | 0.60 | 0.59 | 0.61 | 0.61 | 0.59 |
| 2 | 0.80 | 0.76 | 0.76 | 0.75 | 0.76 | 0.75 | 0.74 |
| Ts2 | 0.87 | 0.84 | 0.84 | 0.84 | 0.84 | 0.84 | 0.83 |
| 3 | 1.21 | 1.11 | 1.11 | 1.11 | 1.12 | 1.11 | 1.10 |
| Ts3 | 1.22 | 1.12 | 1.12 | 1.11 | 1.12 | 1.12 | 1.10 |
| 4 | 1.21 | 1.13 | 1.13 | 1.11 | 1.13 | 1.13 | 1.10 |
| Ts4 | 1.21 | 1.18 | 1.18 | 1.17 | 1.18 | 1.18 | 1.15 |
| 5 | 1.25 | 1.22 | 1.22 | 1.22 | 1.22 | | 1.20 |
| MAD | | 0.056 | 0.059 | 0.067 | 0.057 | 0.064 | 0.081 |
